# Supplementary figures and images for: On the production of ancient Egyptian blue: Multi-modal characterization and micron-scale luminescence mapping
Source: PLoS One. 2020 Nov 24;15(11):e0242549. doi: 10.1371/journal.pone.0242549 (PMC7685487; doi:10.1371/journal.pone.0242549)

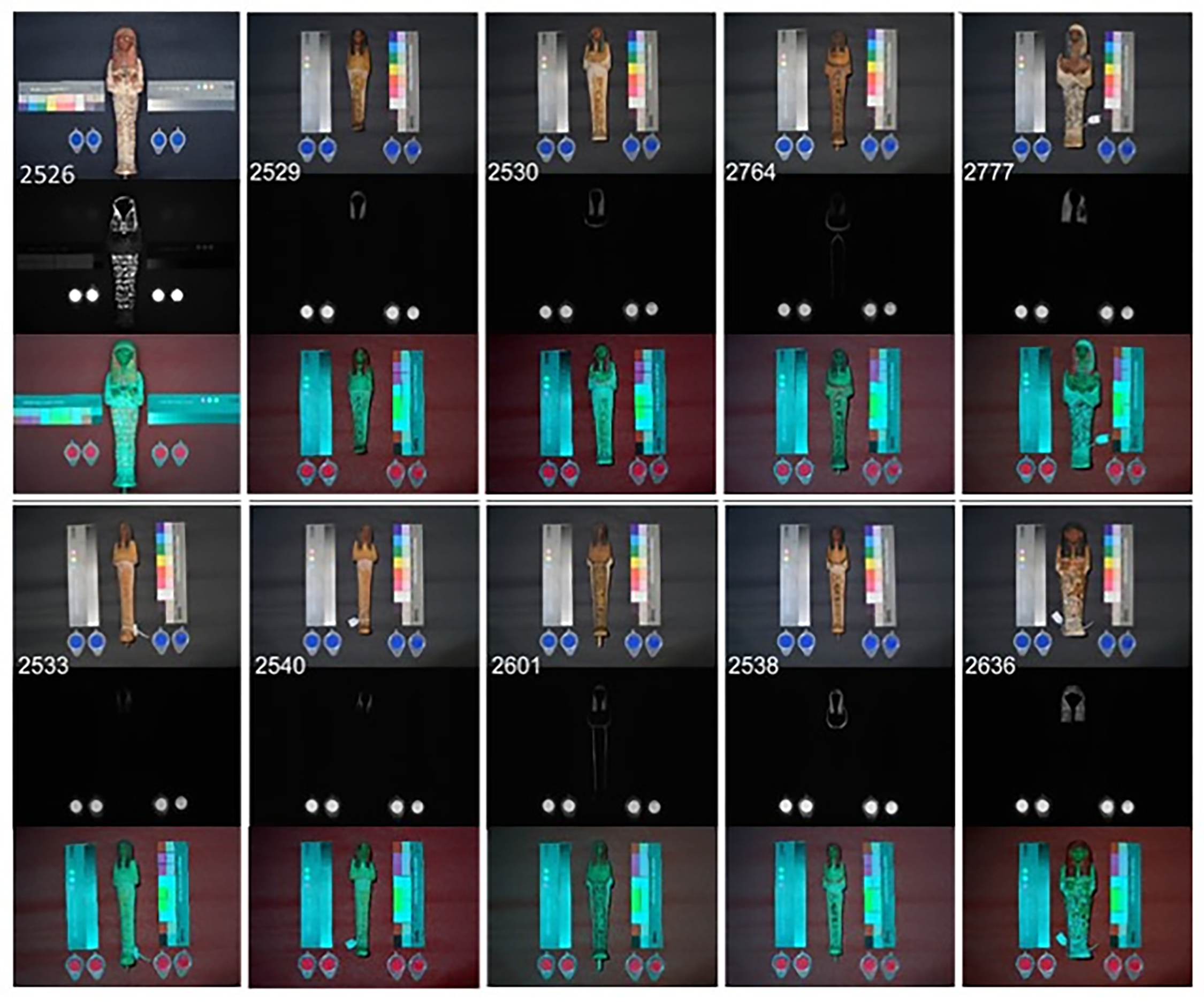

Supplement: S1 Fig — Visible (top), VIL (middle) and VILFC+IRFC (bottom) Imaging of the 10 statuettes from this study. (TIF) [file pone.0242549.s001.tif]

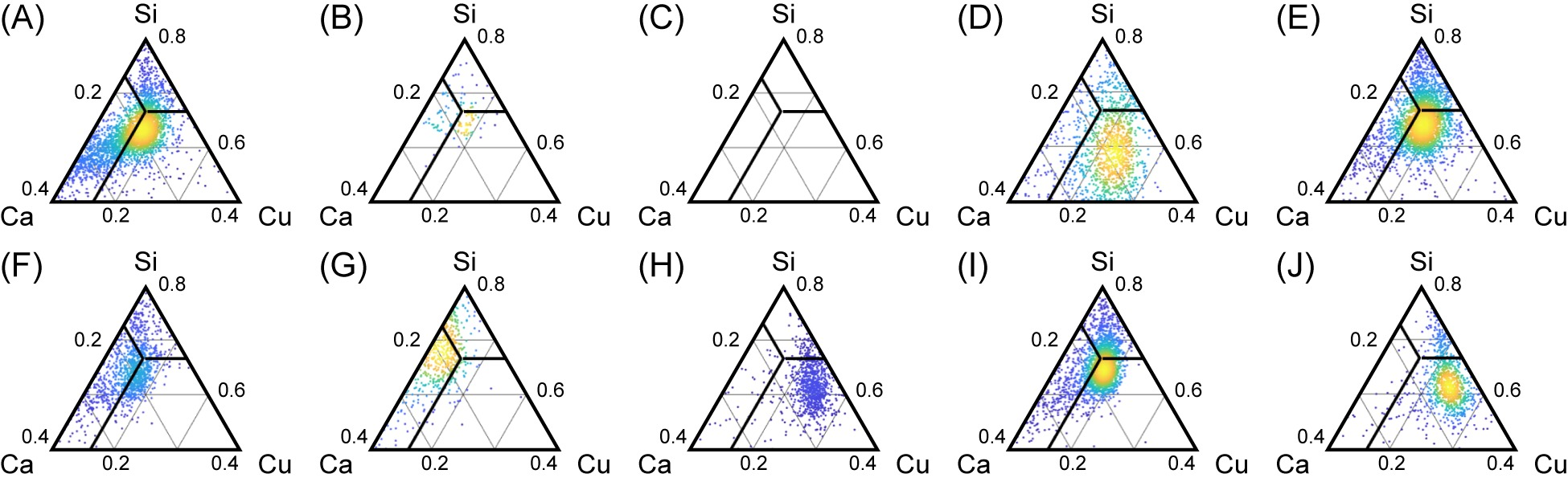

Supplement: S2 Fig — Identities of the samples are (A) 2526; (B) 2529; (C) 2530; (D) 2764; (E) 2777; (F) 2533; (G) 2540; (H) 2601; (I) 2538; (J) 2636. (TIF) [file pone.0242549.s002.tif]

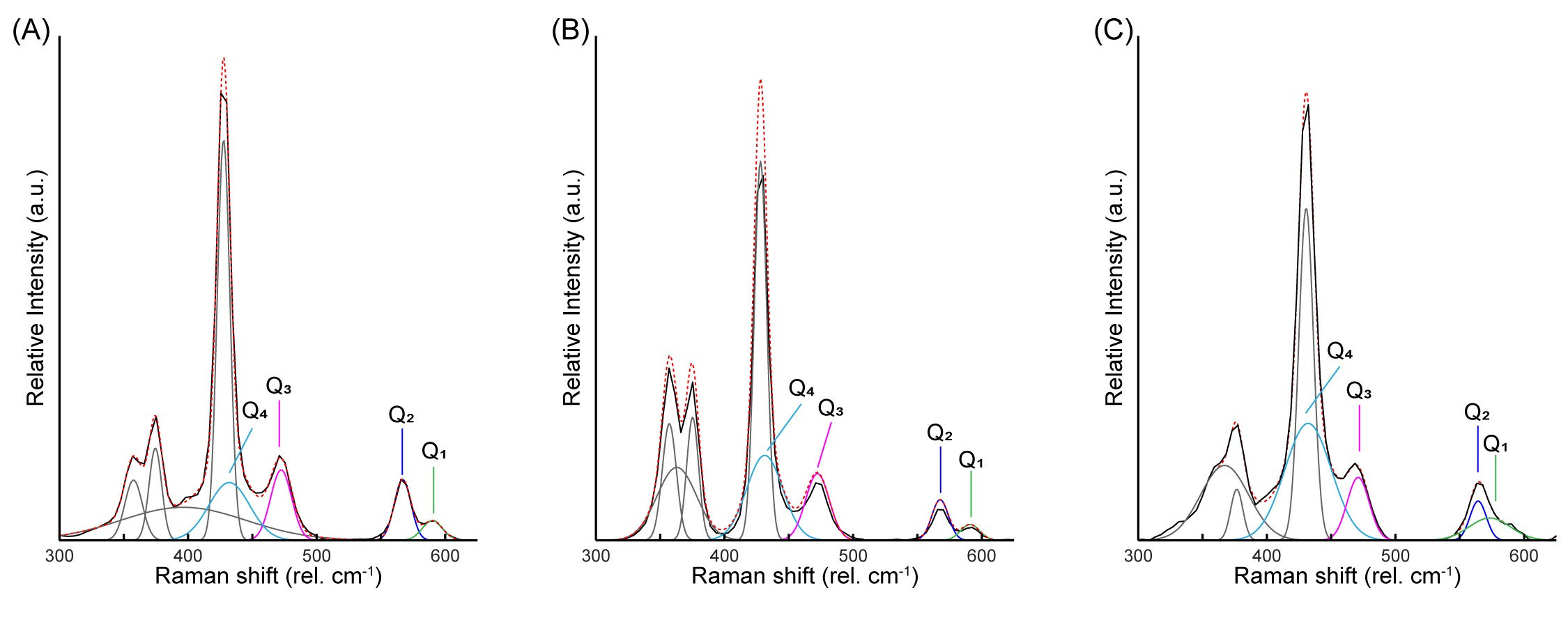

Supplement: S3 Fig — (A) Luminescing phase; (B) non-luminescing phase; (C) modern pigment. (TIF) [file pone.0242549.s003.tif]

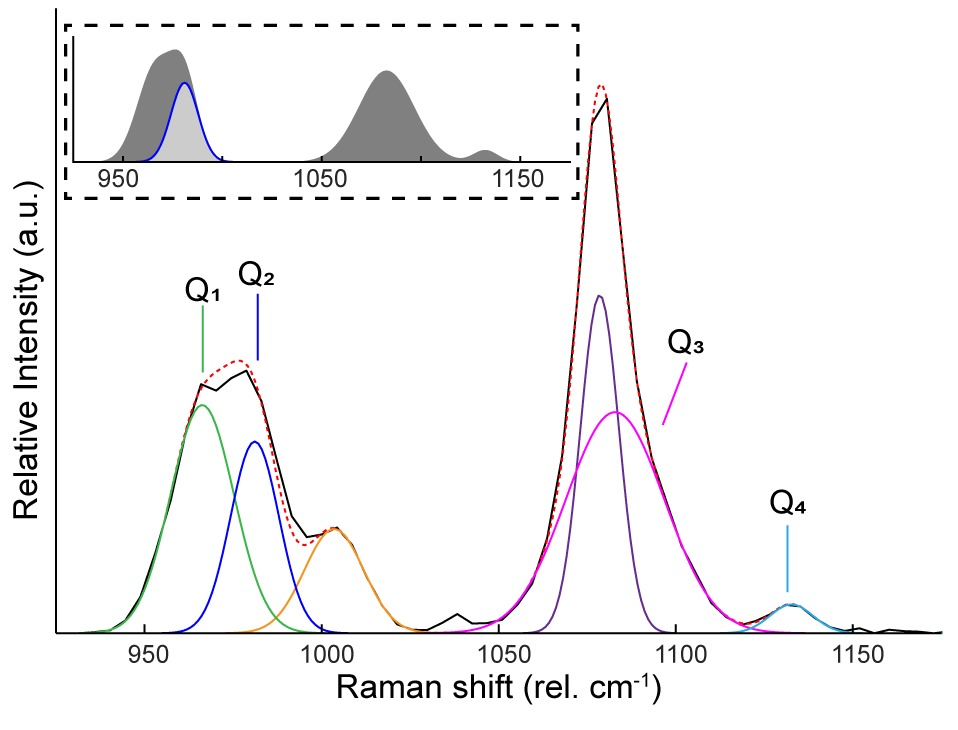

Supplement: S4 Fig — The relative area of the Q2 band (inset) is 0.199 and the approximated index of polymerization is 0.516. (TIF) [file pone.0242549.s004.tif]

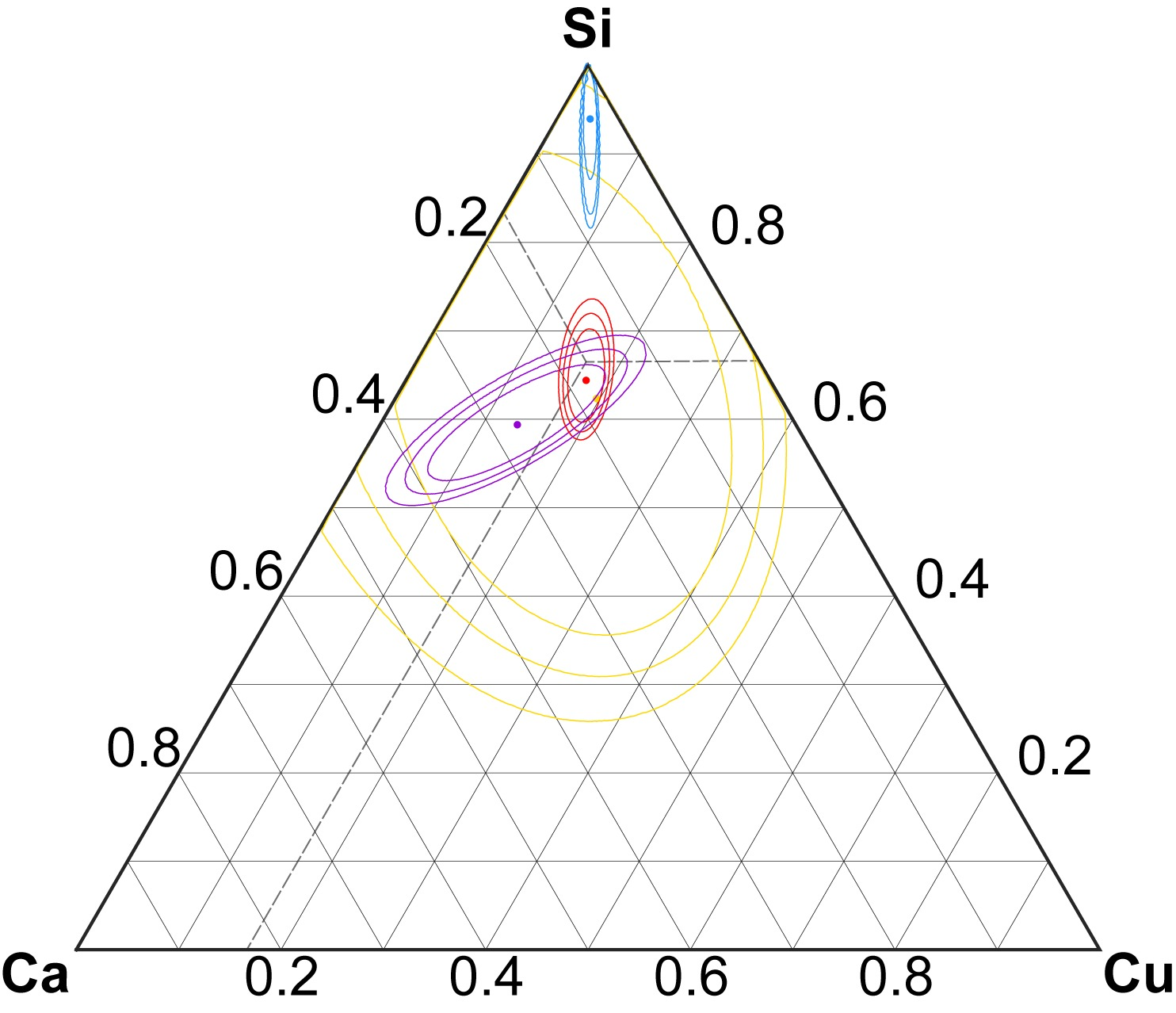

Supplement: S5 Fig — (TIF) [file pone.0242549.s005.tif]

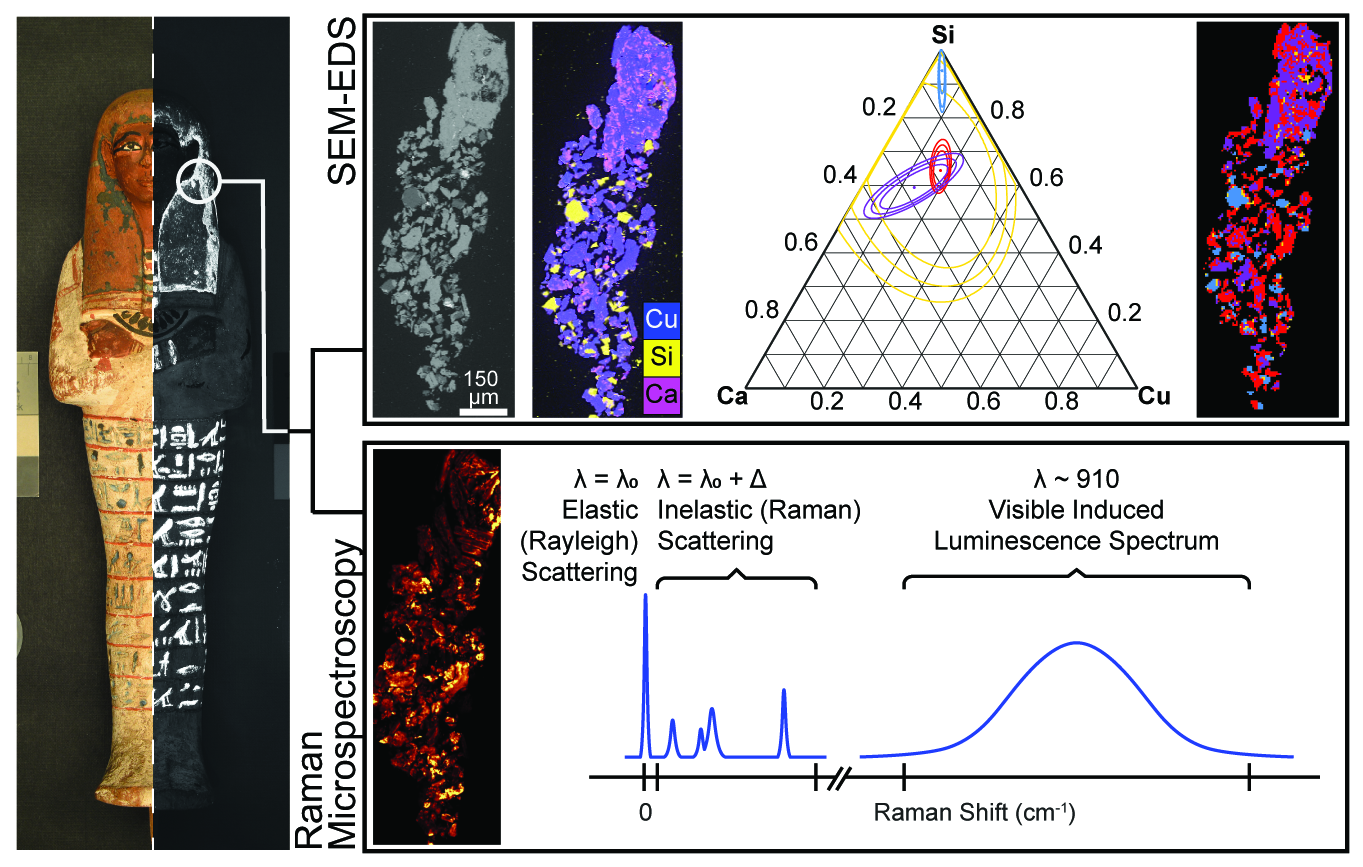

Supplement: S6 Fig — (TIF) [file pone.0242549.s006.tif]
