# Supplementary material for: On the production of ancient Egyptian blue: Multi-modal characterization and micron-scale luminescence mapping
Source: PLoS One. 2020 Nov 24;15(11):e0242549. doi: 10.1371/journal.pone.0242549 (PMC7685487; doi:10.1371/journal.pone.0242549)
Supplement: S1 Table — (DOCX) [file pone.0242549.s007.docx]

**S1 Table.** Sample grouping corresponding to Fig. 2c

| Sample | Average Composition (wt %) | | | Cluster |
| --- | --- | --- | --- | --- |
|  | Ca | Si | Cu |  |
| 2526 | 10.94 | 24.75 | 12.14 | 1 |
| 2538 | 13.55 | 30.85 | 19.89 | 1 |
| 2636 | 5.82 | 24.67 | 14.02 | 1 |
| 2777 | 12.42 | 31.92 | 19.47 | 1 |
| 2529 | 6.80 | 16.56 | 12.13 | 2 |
| 2540 | 11.73 | 12.34 | 4.34 | 2 |
| 2764 | 7.93 | 15.33 | 12.86 | 2 |
| 2530 | 56.22 | 1.29 | 0.22 | 3 |
| 2533 | 34.57 | 14.12 | 5.19 | 3 |
| 2601 | 38.82 | 3.43 | 3.44 | 3 |
