# Supplementary material for: On the production of ancient Egyptian blue: Multi-modal characterization and micron-scale luminescence mapping
Source: PLoS One. 2020 Nov 24;15(11):e0242549. doi: 10.1371/journal.pone.0242549 (PMC7685487; doi:10.1371/journal.pone.0242549)
Supplement: S2 Table — (DOCX) [file pone.0242549.s008.docx]

**S2 Table.** Sample grouping corresponding to Fig. 2f

| Sample | EB Grain Composition (wt %) | | | Cluster |
| --- | --- | --- | --- | --- |
|  | Ca | Si | Cu |  |
| 2526 | 9.50 | 23.99 | 14.81 | 1 |
| 2533 | 9.07 | 19.97 | 9.87 | 1 |
| 2538 | 8.44 | 22.44 | 13.64 | 1 |
| 2777 | 8.32 | 23.45 | 13.41 | 1 |
| 2529 | 3.81 | 10.44 | 6.12 | 2 |
| 2540 | 6.54 | 8.02 | 2.18 | 2 |
| 2601 | 10.50 | 8.33 | 11.68 | 2 |
| 2636 | 3.14 | 14.71 | 7.83 | 2 |
| 2764 | 3.73 | 9.53 | 7.27 | 2 |
| 2530 | 32.82 | 4.77 | 0.13 | 3 |
