# Supplementary material for: On the production of ancient Egyptian blue: Multi-modal characterization and micron-scale luminescence mapping
Source: PLoS One. 2020 Nov 24;15(11):e0242549. doi: 10.1371/journal.pone.0242549 (PMC7685487; doi:10.1371/journal.pone.0242549)
Supplement: Attachment — Submitted filename: Reviewer Comments.docx [file pone.0242549.s009.docx]

PONE-D-20-25163
On the production of ancient Egyptian blue: multi-modal characterization and micron-scale luminescence mapping
PLOS ONE

Dear Dr. Masic,

Thank you for submitting your manuscript to PLOS ONE. After careful consideration, we feel that the manuscript has merit but could be improved to fully meet PLOS ONE’s publication criteria. Therefore, we invite you to submit a revised version of the manuscript that addresses the points raised during the review process.

In particular, please address the comments raised by the Reviewer #2 and make corresponding changes in the manuscript.

Please submit your revised manuscript by Nov 20 2020 11:59PM. If you will need more time than this to complete your revisions, please reply to this message or contact the journal office at [plosone@plos.org](mailto:plosone@plos.org). When you're ready to submit your revision, log on to <https://www.editorialmanager.com/pone/> and select the 'Submissions Needing Revision' folder to locate your manuscript file.

Please include the following items when submitting your revised manuscript:

- A rebuttal letter that responds to each point raised by the academic editor and reviewer(s). You should upload this letter as a separate file labeled 'Response to Reviewers'.
- A marked-up copy of your manuscript that highlights changes made to the original version. You should upload this as a separate file labeled 'Revised Manuscript with Track Changes'.
- An unmarked version of your revised paper without tracked changes. You should upload this as a separate file labeled 'Manuscript'.

If you would like to make changes to your financial disclosure, please include your updated statement in your cover letter. Guidelines for resubmitting your figure files are available below the reviewer comments at the end of this letter.

If applicable, we recommend that you deposit your laboratory protocols in protocols.io to enhance the reproducibility of your results. Protocols.io assigns your protocol its own identifier (DOI) so that it can be cited independently in the future. For instructions see: <http://journals.plos.org/plosone/s/submission-guidelines#loc-laboratory-protocols>

We look forward to receiving your revised manuscript.

Kind regards,

Oksana Ostroverkhova
Academic Editor
PLOS ONE

Journal Requirements:

When submitting your revision, we need you to address these additional requirements.

1. Please ensure that your manuscript meets PLOS ONE's style requirements, including those for file naming. The PLOS ONE style templates can be found at

<https://journals.plos.org/plosone/s/file?id=wjVg/PLOSOne_formatting_sample_main_body.pdf> and

<https://journals.plos.org/plosone/s/file?id=ba62/PLOSOne_formatting_sample_title_authors_affiliations.pdf>

2. Your ethics statement should only appear in the Methods section of your manuscript. If your ethics statement is written in any section besides the Methods, please move it to the Methods section and delete it from any other section. Please ensure that your ethics statement is included in your manuscript, as the ethics statement entered into the online submission form will not be published alongside your manuscript.

[Note: HTML markup is below. Please do not edit.]

Reviewers' comments:

Reviewer's Responses to Questions

**Comments to the Author**

1. Is the manuscript technically sound, and do the data support the conclusions?

The manuscript must describe a technically sound piece of scientific research with data that supports the conclusions. Experiments must have been conducted rigorously, with appropriate controls, replication, and sample sizes. The conclusions must be drawn appropriately based on the data presented.

Reviewer #1: Yes

Reviewer #2: Yes

2. Has the statistical analysis been performed appropriately and rigorously?

Reviewer #1: Yes

Reviewer #2: Yes

3. Have the authors made all data underlying the findings in their manuscript fully available?

The [PLOS Data policy](http://www.plosone.org/static/policies.action#sharing) requires authors to make all data underlying the findings described in their manuscript fully available without restriction, with rare exception (please refer to the Data Availability Statement in the manuscript PDF file). The data should be provided as part of the manuscript or its supporting information, or deposited to a public repository. For example, in addition to summary statistics, the data points behind means, medians and variance measures should be available. If there are restrictions on publicly sharing data—e.g. participant privacy or use of data from a third party—those must be specified.

Reviewer #1: Yes

Reviewer #2: Yes

4. Is the manuscript presented in an intelligible fashion and written in standard English?

PLOS ONE does not copyedit accepted manuscripts, so the language in submitted articles must be clear, correct, and unambiguous. Any typographical or grammatical errors should be corrected at revision, so please note any specific errors here.

Reviewer #1: Yes

Reviewer #2: Yes

5. Review Comments to the Author

Please use the space provided to explain your answers to the questions above. You may also include additional comments for the author, including concerns about dual publication, research ethics, or publication ethics. (Please upload your review as an attachment if it exceeds 20,000 characters)

Reviewer #1: This short paper was enjoyable to read, exciting data, and relevant/refreshing to what I had considered a mature research topic. The micro-spectroscopy of archaeological Egyptian blue (EB) samples from the Egyptian Museum in Torino were studied. The paper's critical insight is that a blue Raman laser (532nm) can both induce the Raman effect and photoluminescence of cuprorivaite in a single experiment, thus acting as a probe to examine grain heterogeneity. Their findings show that, in some cases, regions of an individual EB pigment grain may not luminesce when excited with visible light. This finding could be due to these regions not having formed into a structured crystal or, possibly, due to decomposition due to furnace temperature fluctuations (or excess flux in that area, etc.). I believe more correlative spectroscopy could answer this, and it is a suggestion that the authors look into synchrotron-based micro-diffraction as another way to probe these structures.

These findings lay the ground for experimental replication work into how EB forms and how crystallization occurs out of a flux melt. For the archaeological scientist, such work will help define "workshop practices" of these ancient craftsmen.

We thank the reviewer for their comments and will continue to explore techniques such as micro-diffraction to further expand this work.

Reviewer #2: The paper is a highly challenging analysis of Egyptian artefacts using combined EDX, Raman and micro-luminescence techniques.
The skills of the scientists lead to consistent insights on the nature of luminescence and chemical composition and differences in the pigment layer in the shabti statuette series.

Some clarifications should be implemented in the text:
1. table 1 is almost undescribed, in terms of caption, legend and also underlying method (i.e. IRC is not declared). According to the text, the table could also report samples where traces of heteroelements (K, As, Fe) were found.

Need help from Marco et al on this one

2. lines 113-122: false color images are explained but are not shown neither in the text nor in the SI.

I think I fixed this, but let’s confirm before writing the rebuttal.

3. in figure 2, lines showing the expected cuprorivaite composition could be added (as done in the plots in the SI).

We appreciate this suggestion and have updated the plots accordingly.

4. in figure 2, the "x" of the centroids are poorly visible, and would be useful to make them in the colour referred to the circles they are related with.

We thank the reviewer for this suggestion and have updated the plots to be easier to read.

5. Maybe it is unclear to me, but in the ternary diagram if figure 2d there are 4 gaussian fit distribution but in plot 2f only 3 colored series are shown. Moreover, red circles in 2f are overlapped, I guess...

We have added Tables S1 and S2 in the supplementary materials to clarify that the in 2c and 2f points represent individual samples whereas the Gaussian clustering is for phases within a single sample. The red grouping in Fig 2d has a very low standard deviation so the lines appear overlapping due to the line width, but are distinct ellipses. We have added a supplemental figure of an enlarged figure 2d to clarify this.

6. line 165 on: differences between Raman and photoluminescence are well explained, but in the text (maybe since they are collected with the same equipment) are often used in misleading way. Each time the collected response is the 910 nm response, the technique does not deal with Raman effect but (consistently with the title of the paper) with micron-scale luminescence. The text should be arranged using these guidelines.

We appreciate this observation and agree that we should distinguish between the Raman scattering and observed luminescence. We have updated the text accordingly.

7. line 190: I suppose that the referred resolution is the spatial resolution of 2-4 microns. The notation "per pixel" seems to me uncorrect.

We thank the reviewer for this comment and have reworded the sentence accordingly.

6. PLOS authors have the option to publish the peer review history of their article ([what does this mean?](https://journals.plos.org/plosone/s/editorial-and-peer-review-process#loc-peer-review-history)). If published, this will include your full peer review and any attached files.


If you choose “no”, your identity will remain anonymous but your review may still be made public.


**Do you want your identity to be public for this peer review?** For information about this choice, including consent withdrawal, please see our [Privacy Policy](https://www.plos.org/privacy-policy).

Reviewer #1: No

Reviewer #2: No

[NOTE: If reviewer comments were submitted as an attachment file, they will be attached to this email and accessible via the submission site. Please log into your account, locate the manuscript record, and check for the action link "View Attachments". If this link does not appear, there are no attachment files.]

While revising your submission, please upload your figure files to the Preflight Analysis and Conversion Engine (PACE) digital diagnostic tool, <https://pacev2.apexcovantage.com/>. PACE helps ensure that figures meet PLOS requirements. To use PACE, you must first register as a user. Registration is free. Then, login and navigate to the UPLOAD tab, where you will find detailed instructions on how to use the tool. If you encounter any issues or have any questions when using PACE, please email PLOS at [figures@plos.org](mailto:figures@plos.org). Please note that Supporting Information files do not need this step.
